# Graphene Oxide Deposited with Transition Metal Chalcogenide for Selective Extraction and Determination of Hg(II): Experimental and Computational Analysis

**DOI:** 10.3390/nano13010137

**Published:** 2022-12-27

**Authors:** Huda Alsaeedi, Hilal Ahmad, Malak Faisal Altowairqi, Nouf AbdulRahman Almuryyi, Ali Alsalme

**Affiliations:** 1 Department of Chemistry, College of Science, King Saud University, Riyadh 11451, Saudi Arabia; 2 Division of Computational Physics, Institute for Computational Science, Ton Duc Thang University, Ho Chi Minh City 700000, Vietnam

**Keywords:** graphene oxide, mercury, water analysis, solid phase extraction

## Abstract

A graphene oxide (GO/CdS) nanocomposite was synthesized by an in situ hydrothermal process and studied to develop a micro solid phase extraction procedure. Microscopic and spectroscopic characterizations have confirmed the successful preparation of the GO/CdS composite. The prepared nanocomposite selectively extracts Hg(II) ions from various water samples (tap, river, and groundwater). The intriguing characteristic of GO sheets is to provide exceptional hydrophilicity and Hg(II) accessibility to surface-decorated CdS nanoparticles. The GO/CdS nanocomposite shows excellent extraction of trace Hg(II) in a short interval of time. Computations based on density functional theory (DFT) suggest that energetically favorable multinuclear S-Hg binding leads to rapid adsorption with high sorption capacity at GO/CdS sites. The analytical features of merit suggested that the developed method has a low detection limit (0.07 µg L^−1^) and shows good accuracy and precision (with RSD 3.5%; N = 5). The developed method was verified by analyzing SRM 1641d (Standard Reference Material) and real samples after spiking to a predetermined amount.

## 1. Introduction

Mercury is a toxic element and is usually found in the soil as well as in groundwater [1,2,3]. It can therefore readily permeate into living organisms through the food chain [4,5]. However, it is not among the essential elements required for metabolic activities, and therefore has a profoundly damaging effect on the biota [6]. Inorganic mercury ions (Hg(II)) and their complexes are frequently found in the environment and cause severe illness in humans [7]. The release of Hg(II) into water streams by anthropogenic activities has led microorganisms and aquatic creatures (shellfish and fish) to bioaccumulate it and pass it to humans through the food web and food chain [8,9]. Therefore, consumers like humans are at great risk of increased mercury exposure because of global consumption of contaminated seafood and farm-cultivated rice [5,9,10]. Moreover, Hg(II) in polluted ground waters can also be accumulated by the plant roots of different species, several of which are edible.

The safety guidelines drawn up by the European Communities determine an acceptable weekly total Hg consumption of 1.6 g kg^−1^ of body weight, as laid out in Regulation (EC) No 1881/2006 [11], while WHO (World Health Organization) has set an alarming value of 6 µg L^−1^ of Hg concentration in drinking water [12]. Considering this, it is of high value to regularly monitor the Hg(II) concentrations in consumable water and seafood, which are possibly contaminated with traces of Hg(II). However, directly determining the contaminants in real samples with high accuracy, using atomic absorption (FAAS) or plasma spectrometric techniques (ICP-OES or ICP-MS), is challenging due to complex sample matrices and the high salt composition of samples causing substantial spectral interferences in analyte determination [13,14,15,16]. Because Hg(II) is usually found at trace and ultra-trace levels and may be associated with complex sample matrices, many instrumental detection limits (LOD) may be inadequate or substantial interference may occur due to the complex matrix [17,18]. As a consequence, sample preparation or preconcentration techniques are frequently desired to execute the separation of the analyte from a complex matrix [19,20].

Solid phase extraction (SPE) is the most suitable analyte extraction technique gaining popularity as a possible alternative to liquid extraction techniques. SPE has several advantages, such as a high preconcentration factor, good repeatability, cheap operational cost, minimal or no use of organic solvents, and the ability to combine SPE with spectroscopic methods [21,22]. Furthermore, if an appropriate solid adsorbent is utilized, SPE allows for the selective identification of analytes. For this purpose, the preparation of new materials for the extraction and preconcentration of Hg(II) might be useful in trace analysis. Traditional and novel mercury adsorbents, such as molybdenum disulfide nanocomposite [23,24], activated carbon [25,26], functionalized membranes [27], metal–organic frameworks [28], and ionic liquids [29] have been studied so far. However, with a high-salt matrix, many of the prepared adsorbents lack selectivity for Hg(II). As a result, their use in conjunction with other spectroscopic methods would be limited due to the complicated matrix and/or high salinity of the samples. Hg(II) may also be found in acidic conditions, such as sludges and wastewater. As a result, efficient adsorption of Hg(II) ions under the aforementioned circumstances is desirable, as it can greatly simplify and reduce sample preparation time.

Herein, we prepared a CdS (cadmium sulfide)-nanoparticle-decorated GO (graphene oxide) surface using a hydrothermal synthetic strategy. Later, the adsorption of Hg(II) ions on GO/CdS was considered, and its good selectivity characteristics were discovered. In acidic samples (pH 4.5), the GO/CdS adsorbent quantitatively adsorbed Hg(II) with a maximum adsorption capacity of 280 mg g^−1^ and a relatively low adsorbent dose (5 mg in 100 mL of the sample). Furthermore, GO/CdS has a high selectivity for Hg(II) ions compared to other divalent ions. Other coexisting ions have no effect on the recovery of Hg(II) ions up to a 1000-fold excess. The formation of exceptionally stable complexes of Hg(II) with GO/CdS leads to the strong selectivity of GO/CdS toward Hg(II) ions, which might be due to the hard-soft-acid-base hypothesis [30,31]. The Soft S-donor atom in CdS nanoparticles forms more stable complexes with the soft Hg(II) cation than other co-ions existing together, which were discussed in later sections [31]. The developed approach is reliable, quick, and accurate in detecting trace Hg(II) ions in complicated samples. These findings reveal that GO/CdS may be used in micro amounts in micro SPE due to its strong adsorption capacity toward Hg(II) ions and superior dispersibility in aqueous samples, due to a high number of oxygen-containing functional groups in GO solid support. The methodology established here is used to examine a variety of samples, including food and drinking water specimens.

## 2. Experiment

### 2.1. Materials and Chemicals

Merck Millipore provided a standard solution of mercury nitrate (1 mg L^−1^) and nitrocellulose membranes 450 µm in thickness (Burlington, MA, USA). Nitric acid and sodium hydroxide were acquired from Sigma Aldrich to make pH adjustments to the solutions (St. Louis, MO, USA). The tests were conducted with ultrapure water provided by a Milli-Q system (Millipore, Gmbh, Germany). The SRM 1641d obtained from NIST was used after appropriate dilution for method validation.

### 2.2. Instruments

A field emission scanning electron microscope (FESEM, SIGMA, ZEISS, Blackwood, NJ, USA) was used to obtain the micrographs. A tunneling electron microscope (TEM, TECHNAI G2 30S Twin instrument, FEI, Hillsboro, Oregon, USA) was used to observe the high-resolution micrographs of the sorbent. The zeta potential (z) measurements to observe the surface charge of the sorbent were investigated using Zetasizer (Malvern Instruments, Gmbh, Kassel, Germany). X-ray photo electron spectra were recorded using XPS, ThermoFisher, ESCALABA 250XI instrument, Waltham, MA, USA. FTIR spectra were recorded on a Bruker Spectrometer, Waltham, MA, USA. The X-ray diffraction pattern was obtained on an X-ray diffractometer (Smart Lab XRD, Rigaku, New Delhi, India) with Cu Kα radiation. An inductively coupled plasma optical emission spectrometer (ICPOES, Perkin Elmer Avio 200, Waltham, MA, USA) was used to measure the metal ion concentration.

### 2.3. Synthesis

**Figure 1:**
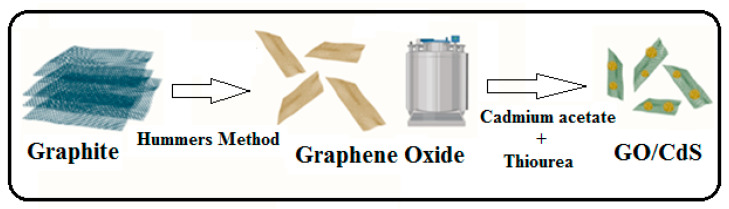
Schematic illustration of GO/CdS synthesis steps.

The modified Hummers’ approach was used to synthesize the GO for further modification [32]. The GO/CdS composite was prepared by the in situ hydrothermal method. Briefly, 2 g of GO was dispersed in 50 mL of deionized water (DW) and probe-sonicated for 2 h at 27 °C. A total of 0.5 g of cadmium acetate was added to the GO suspension and mixed well. To this solution, 0.3 g of thiourea was gently added, and the whole reacting mixture was vigorously mixed and transferred to a hydrothermal assembly (Teflon-lined). The hydrothermal setup was kept in an air oven at 180 °C for 12 h. After cooling, the resultant suspended solid particles were centrifuged and washed repeatedly with ethanol and deionized water before being dried in a vacuum oven at 60 °C for 6 h. The produced GO/CdS powder was characterized, and Hg(II) adsorption was investigated; Figure 1 illustrates the synthesis steps.

### 2.4. Preconcentration Procedure of Hg(II) Ions

The prepared nanocomposite GO/CdS was dispersed in DW (1 mg mL^−1^) in the first phase to produce a homogeneous suspension. The GO/CdS suspension was then added to 50 to 100 mL of the tested samples (model) maintained at pH 4.5. After that, the sample solution was agitated for 10 min for complete Hg(II) adsorption. After this period, the solution was filtered off using the nitrocellulose membrane. The collected GO/CdS with adsorbed Hg(II) ions was agitated with 5 mL of 0.1 M HCl acid, and the collected eluent was analyzed directly by ICP-OES.

## 3. Results and Discussion

### 3.1. Characterization

**Figure 2:**
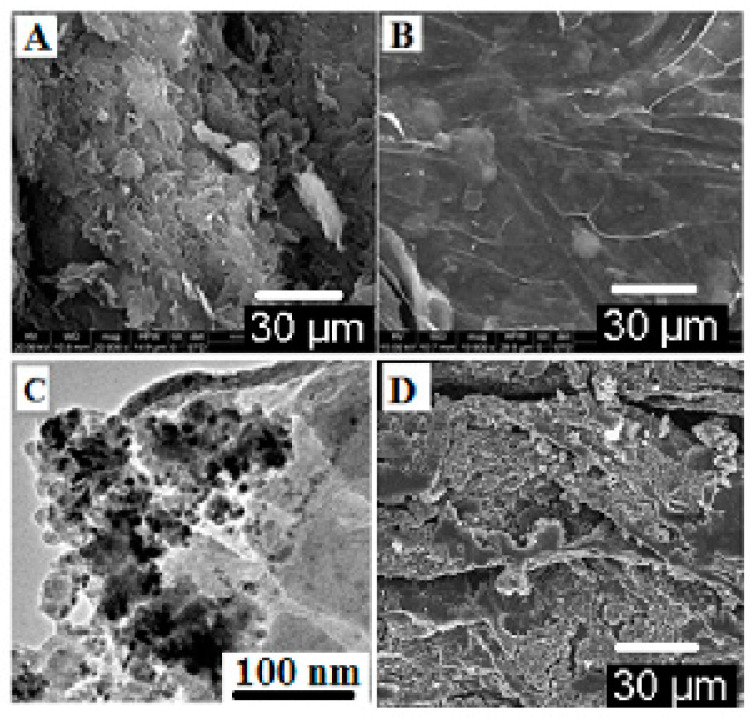
FESEM images show the surface morphology of (**A**) nascent graphene oxide and (**B**) GO/CdS; (**C**) TEM image of GO/CdS and (**D**) FESEM image of GO/CdS after Hg(II) sorption cycle.

A SEM was used to observe the surface of the nascent GO and GO/CdS composite (Figure 2A,B). In Figure 2A, it can be seen that the surface of the nascent GO sheets was smooth with textural wrinkles. On the other hand, after functionalization, the decoration of CdS particles is readily visible on the surface of the GO/CdS sheets, as shown in the SEM image in Figure 2B. Furthermore, TEM observations clearly indicate the decoration of CdS nanoparticles on the GO surface (Figure 2C). These observations suggest that the CdS particles had been successfully decorated on the GO surface. Figure 2D represents the SEM image after Hg(II) adsorption.

**Figure 3:**
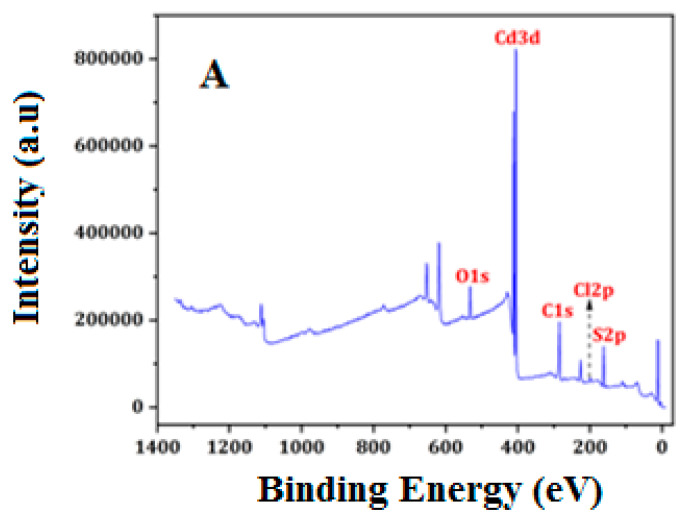

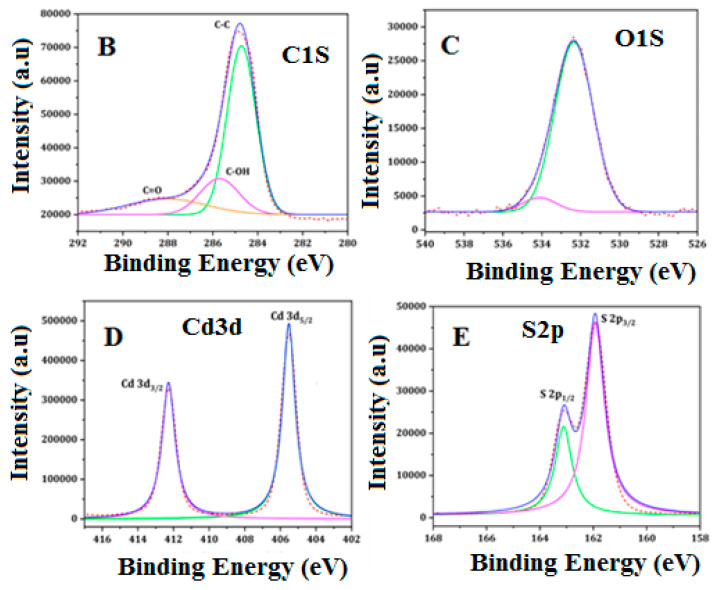
XPS: (**A**) Complete survey, deconvoluted spectra. (**B**) C 1s, (**C**) O 1s, (**D**) Cd 3d_5/2_, and (**E**) S 2p_3/2_.

Utilizing XPS data, we investigated the GO/CdS composite’s surface components (Figure 3A). The data presented in Table 1 provide the binding energies with percent composition for C and O of the GO sheets and Cd and S of the deposited CdS of GO/CdS. Two main peaks were observed for GO; the peak at 284.6 eV was assignable to the sp^2^-carbon species, while the peak at higher binding energies (285–288 eV) was ascribed to oxygenated carbon species, such as hydroxyl and carboxyl groups (Figure 3B). The binding energies of Cd 3d_5/2_ were found at 405.5–412.2 eV (Figure 3D), suggesting that cadmium was in the Cd^2+^ state. The S 2p_3/2_ peaks were observed at 161.1–163.1 eV (Figure 3E), in agreement with the expectation that sulfur existed as the sulfide species (S^2−^). These observations take into account the structural composition of the prepared sorbent material.

**Figure 4:**
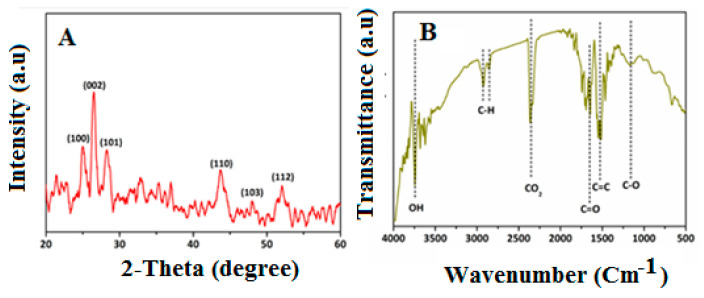
(**A**) XRD pattern and (**B**) FTIR spectra of CdS-GO nanocomposite.

XRD analysis was used to examine the nanocomposites’ crystalline structures. The strongest peak, which matched the peak of graphene, was seen in (002), while the remaining peaks were matched to CdS nanoparticles. In fact, the major peaks of the CdS-graphene nanocomposite were also detected in the XRD pattern, which was further supported by the existence and purity of the graphene. This proves that the CdS component in the GO/CdS composites is formed into a pure hexagonal phase with good crystallinity, since the diffraction peaks of the GO/CdS composites are well indexed to crystal planes of a hexagonal-phase CdS (Figure 4A). According to the above FTIR data (Figure 4B), GO contains a large number of polar functional groups The oxygen-containing functional groups of GO were revealed by the bands (Figure 4B), which correspond to C–O stretching vibrations, C–OH stretching peak, C–O–H deformation peak, and C–O stretching of COOH groups, respectively.

### 3.2. Effect of Solution pH

**Figure 5:**
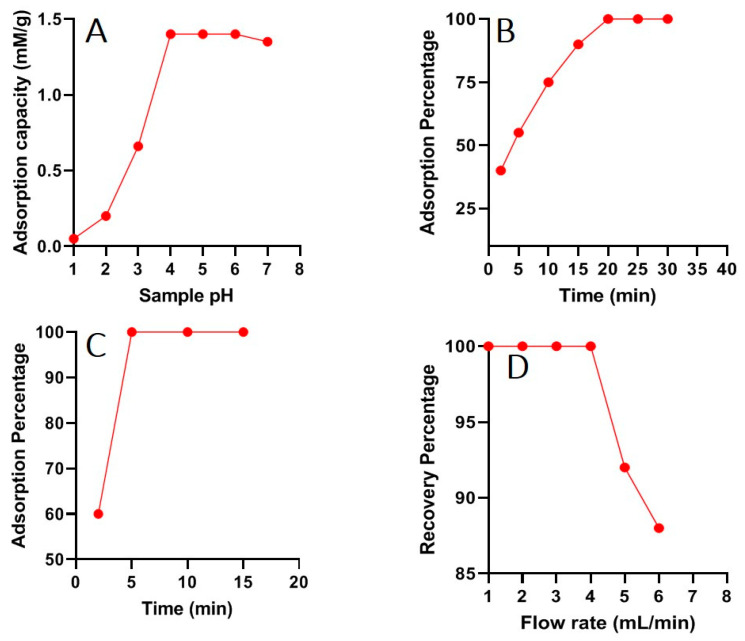
(**A**) Effect of solution pH on the sorption of Hg(II) (sample volume 50 mL; metal ion 100 mg L^−1^; contact time 30 min; sorbent amount 25.0 mg); (**B**) Effect of sample volume on the preconcentration on Hg(II) at higher sample volume (500 mL) and (**C**) at lower sample volume (100 mL) (metal ion 1 mg L^−1^; sample pH 5.0; sorbent amount 25.0 mg); (**D**) Effect of sample flow rate on the recovery of Hg(II) (sample volume 100 mL; metal ion 1 mg L^−1^; sample pH 5.0; sorbent amount 25.0 mg).

The sample pH has a vital effect in the adsorption process because it affects the adsorbent surface charge present in the solution; therefore, studying the effect of sample pH is particularly significant in the SPE. In the pH range of 1 to 7, the influence of sample pH on Hg(II) adsorption was explored. Figure 3 shows the obtained results for Hg(II) adsorption. It was observed that at lower pH values, the adsorption of Hg(II) was less and increased rapidly after pH 4 (Figure 5A), and the maximum adsorption of Hg(II) was attained at pH 4–5. The selective adsorption of Hg(II) was observed due to chelation via −S active sites onto GO/CdS surfaces. At pH 2–3, the protonation of active sites generates a positive charge on the surface of GO/CdS; as a result, there is less physio-chemical interaction between the GO/CdS and Hg(II). The zeta potential value observed for the GO-CdS composite was found to be 4.2. As a result, the sample pH increases from pH 4 onwards, due to the deprotonating of surface groups, the GO/CdS gets a negative surface charge at neutral to alkaline pH, and the electrostatic contacts between Hg(II) cations and GO/CdS nanosheets grow stronger, leading to high adsorption capacity [32,33]. Furthermore, adsorption by chelation is a primary force, in addition to electrostatic interactions. The −S active sites of GO/CdS with adjacent carboxyl and hydroxyl groups could form a chelate with the Hg(II) ions. In the case of oxyanions, another phenomenon can be observed. The interaction between the coexisting anions and the functional groups (deprotonated) of the GO/CdS is not conceivable in a neutral to basic solution [34,35]. Because of their negative surface charge, the anions cannot be accommodated by the deprotonated functional groups of GO/CdS at pH > 4, thus decreasing the interference by rejecting the anions of the sample solution.

### 3.3. Theoretical Calculations

**Figure 6:**
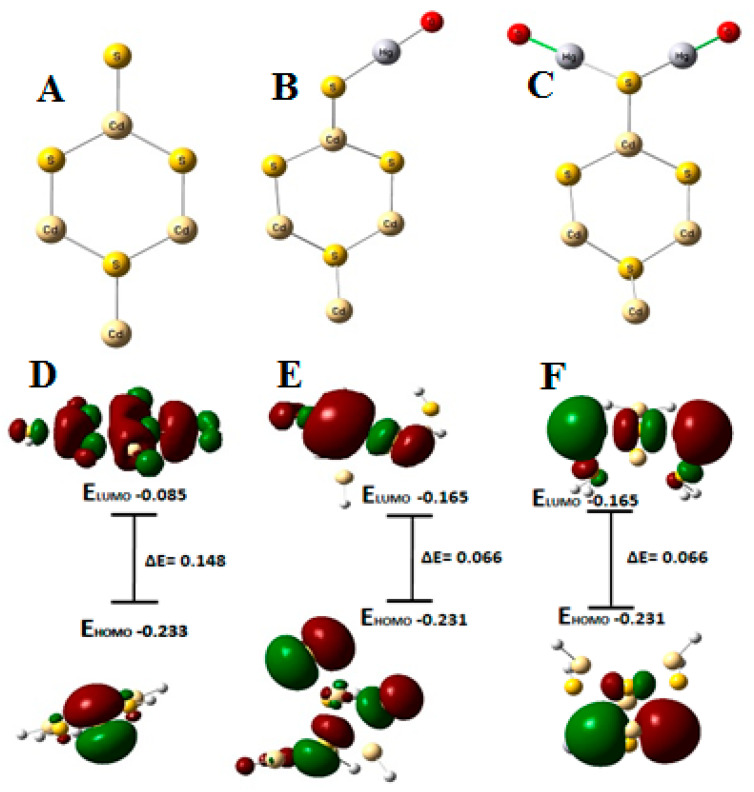
Optimized geometries of (**A**) CdS, (**B**) CdS-Hg, and (**C**) Hg-CdS-Hg binuclear complex; (**C**–**F**) are energy diagrams and Kohn–Sham frontier molecular orbitals of CdS, CdS-Hg, and Hg-CdS-Hg, respectively.

The spin-polarized density-functional-theory (DFT)-level computations were performed by G09 Simulation Package (Gaussian, version 09, revised) using the B3LYP hybrid functional [36]. During geometry optimization, there were no symmetry limitations enforced. Analytical calculations of the Hessian matrix were made for the optimized structures to confirm the placement of the proper minima. The binding energy of single and polynuclear Hg(II) ions at the surface of CdS was calculated using a single CdS cell (Figure 6). To determine the Hg binding energy, Ebind, the Hg(II) ions were attached sequentially at the -S site of the CdS surface. The findings demonstrate that the initial Hg(II) has a binding energy of −1.38 eV. Additionally, the Ebind increases for the capture of the second Hg and was discovered to be −5.60 eV for the second ion adsorption. With an Ebind of −1.38 eV and −5.60 eV, for single and double Hg(II), respectively, we discovered that the -S group firmly binds to the binucleated Hg(II) clusters. The results of the DFT calculations imply that the Hg ion binding at the CdS surface was endothermic and energetically more favorable for multinuclear binding. The presence of the driving force of multiple adsorptions, which would have provided an exothermic energetic trend, fit the pattern in the polynuclear distribution of Hg(II) over the CdS adsorbent in the total adsorption capacity that was experimentally observed.

An essential tool for describing a molecule’s interaction is the examination of its highest occupied molecular orbital (HOMO) and lowest unoccupied molecular orbital (LUMO). EHOMO denotes a molecule’s capacity to transfer electrons to an appropriate receptor, whereas ELUMO denotes a molecule’s capacity to accept electrons. A molecule with a low energy gap, such as ΔE (ELUMO-EHOMO), often has a high level of chemical reactivity. Figure 6 shows the diagrams of the Hg-CdS complexes’ frontier molecular orbitals for both mono- and binuclear complexes. The picture demonstrates that HOMOs are dispersed on the sulfur atoms in [Hg-CdS] complexes, but LUMOs are mostly on the Hg. It is noteworthy that the energy band gap of mononuclear HOMO and LUMO was lowered to 0.066 eV, irrespective of CdS (0.148), and was consistent upon binuclear complexation with the Hg, suggesting favorable adsorption. No change in the energy band gap and HOMO LUMO energy levels upon the addition of the second Hg(II) ions was observed; this suggests that the second Hg(II) most probably adsorbed by a physical adsorption (electrostatic attraction) instead of a chemical adsorption (ionic bond formation).

### 3.4. Effect of Sample Volume and Contact Time

At a fixed Hg(II) concentration of 1 mg L^−1^, it was observed that with higher sample volumes (500 mL) and short contact durations (5–20 min), the recovery values for Hg(II) were reduced to 85% of total feed concentration. In order to complete the adsorption of Hg(II) from a sample volume of 500 mL, at least 30 min of sorption time is necessary (Figure 5B,C). To summarize, in order to acquire the best results in the minimum time duration of 5 min, the sample should not exceed 100 mL for Hg(II) determinations. Complete Hg(II) recoveries and low detection limits can be attained in a short duration of time by keeping low sample volumes. Because of its wide surface area and high water dispersibility, the GO/CdS nanocomposite makes excellent contact with the investigated solution, allowing for rapid adsorption of Hg(II) ions and the establishment of an equilibrium state (in less than 5 min, as previously mentioned).

Moreover, we investigated the Hg(II) adsorption in flow-through circumstances. The GO/CdS nanocomposite (25 mg) was deposited on nitrocellulose membranes using vacuum filtration, the obtained membrane was packed in a short glass column (8 × 1 cm), and the model solution (100 mL and 1 mg L^−1^) was subsequently passed through the membrane filter at a flow rate of 4 mL min^−1^. The sorbed metal ion was eluted using 5 mL of eluent (1 M HCl) and subsequently analyzed by ICP-OES. Figure 5D shows the effect of sample flow rate on Hg(II) recovery. The results show that the adsorption of Hg(II) achieves a maximum of 100% and remains consistent across the whole flow rate range of 1–4 mL min^−1^. Afterward, at a flow of 5 mL min^−1^, an 8% fall in Hg(II) recovery was discerned. In conclusion, GO/CdS may be employed successfully in both dispersion and flow settings, and the sorption process is very quick in both cases.

### 3.5. Eluent Effects and Reusability

The sorption capacity and quantitative recovery of sorbed analyte are the two critical characteristics of an excellent sorbent. Elution tests for GO/CdS were carried out using HCl and HNO_3_ in various volumes (1–8 mL) and concentrations (0.05–1.0 M). A total of 5.0 mL of 0.1 M HNO_3_ might provide a maximum recovery of 92% among them. On further increasing the eluent volume to 8 mL, not much difference in the recovery of Hg(II) was observed (95% recovery). When 5 mL of 0.1 M HCl was utilized, nearly full desorption of Hg(II) was found (recovery > 99%). At various volumes, the effectiveness of 0.1 M HCl was investigated (1–10 mL). It was discovered that less than 5 mL volume of 0.1 M HCl acid was ineffective for quantitative recovery of Hg(II) from the sorbent (3 mL gives only 89% recovery). For subsequent research, 5 mL of 0.1 M HCl was utilized for elution. Furthermore, it was found that during the stripping step of Hg(II), there was no significant leaching of CdS nanoparticles from the GO surface. As the eluent acid content was determined by ICP-OES, there was no seeping off of CdS from GO/CdS. Moreover, the recovered sorbent may be reused four times without losing capacity. Following that, a progressive reduction in capacity was noted, culminating in an 8% loss of analyte uptake in the fifth cycle. This might be due to the loss of CdS particles from the GO surface after repeatedly treating with 0.1 M HCl eluent, since we observed traces of Cd in the eluent in the fifth cycle. As a result, the GO/CdS may be reused for up to four consecutive rounds in the separation/preconcentration of Hg(II) from environmental samples.

### 3.6. Study of Coexisting Ions including Humic Acid and Other Organic Acids

The influence of alkali and alkaline earth metals, anions, and organic acids has been studied on the recovery of Hg(II) using the GO/CdS sorbent. Foreign ion concentrations, including humic and fulvic acid with varying concentrations, have been studied [33]. The obtained results presented in Table 2 show that the presence of alkali and alkaline elements and common heavy metal ions had no effect on the sorption of the Hg(II) up to a certain tolerance level. The recoveries for Hg(II) were all above 95% in the presence of these cations and corresponding anions. The recovery of Hg(II) ions can be hampered by the presence of humic and fulvic acid in the water sample at concentrations of 50 mg L^−1^ and 38 mg L^−1^, respectively. Finally, the presence of PO_4_^3−^ and SO_4_^2−^ at high concentrations (500 mg L^−1^) affects Hg(II) recovery marginally. Since SO_4_^2−^ concentrations in river and lake water can reach many tens of milligrams per liter, the PO_4_^3−^ and SO_4_^2−^ content in drinking water does not exceed 250 mg L^−1^. As a result, high levels of these anions should not be a concern in water analysis for Hg(II) determination.

### 3.7. Analytical Figures of Merit

The various analytical figures of merit (calibration, precision, limit of detection (LOD), accuracy, and robustness) of the devised process using GO/CdS as a solid sorbent and ICP-OES analysis has been studied. The Hg(II) analyte in the examined concentration range had high linearity of 2–500 µg L^−1^, with a correlation coefficient of R^2^ = 0.9998. It should be emphasized that the Hg(II) was preconcentrated from varying sample volumes and with a wide concentration range as a micro solid phase extraction technique for ICP-OES analysis. As a result, the matrix effects were eliminated, resulting in a linear connection between emission intensity and analyte concentration. The LOD for Hg(II), estimated as 3 S/m of mean blank signal for 10 replicate measurements, was found to be 0.07 µg L^−1^. As a result, they are suitable for analyzing water samples in accordance with USEPA rules. All of the Hg(II) analytes examined had adequate precision, with RSD values ranging from 1.2 to 4.5%. The established approach is useful for the examination of a wide range of biological and environmental components, taking into account both LODs and accuracy.

### 3.8. Application

The analytical procedure’s reliability was first tested by analyzing SRM and by spiking various real water samples with Hg(II) at various known concentration levels. The obtained findings (Table 3 and Table 4) show that the Hg(II) recoveries were acceptable (in the range of 95–100%) and therefore adequate for the identification of analytes at trace levels in this kind of water sample. The average RSD value characterizing the precision of the suggested method was around 4%, which was satisfactory for the proper application. As a result, the developed MSPE technique, which uses GO/CdS as a sorbent, may be utilized to determine trace amounts of Hg(II) in water samples. Analysis of Standard Reference Material seawater confirmed the correctness of the analytical process. The certified values for Hg were in good agreement with the findings obtained for SRM 1641d (Table 3). The results in Table 3 demonstrate that the suggested approach can successfully identify Hg(II) in water samples. A reasonable consistency between the added and determined amounts of analytes confirms the efficacy of the devised process for the extraction and preconcentration of trace concentrations of Hg(II).

## 4. Conclusions

The prepared GO/CdS nanomaterial combines the outstanding properties of GO, such as good dispersibility in aqueous solutions, with the great affinity of the CdS molecule for Hg(II) ions, for the detection of trace levels of Hg. A comparison of GO/CdS with other sorbents has been made and presented in Table 5. It can be demonstrated that mercury ions can be removed with a very low GO/CdS dosage (5 mg L^−1^). Furthermore, the adsorption time is quite low, ranging from 10 to 30 min for adsorption of 95–100% Hg(II), due to the great dispersibility of GO/CdS nanosheets in aqueous solutions. The selectivity of the GO/CdS toward Hg(II) is exceptional due to the establishment of a strong S–Hg interaction. With high ionic strength and high concentrations of possibly coexisting ions, Hg(II) ions can be removed from aqueous solutions evenly. Hg(II) ions from mildly acidic solutions can be adsorbed quantitatively on the GO/CdS, which is unusual. The acidic media prevent the precipitation of heavy metal ions, which are prevalent in real-world samples. The developed MSPE/ICP-OES approach has detection limits that are lower than the maximum contamination levels for Hg(II) that can be detected in drinking water, as specified by USEPA and WHO guidelines. The combination of MSPE and ICP-OES analysis is extremely advantageous. Because GO/CdS is selective for Hg(II), it could be utilized to analyze Hg(II) in a high-salt matrix with high accuracy and without causing spectral interference.

## Figures and Tables

**Table 1:** XPS data of the GO/CdS sorbent.

Element	Peak Position (eV)	FWHM	Area (P) cps	Atomic %
C 1s	284.8	1.77	128,088	55.82
O 1s	532.38	2.43	76,245	13.75
S 2p	161.92	1.03	72,277	15.53
Cd 3d	405.52	1.00	914,344	14.90

**Table 2:** Effect of Foreign Ions on the Recovery of Hg(II).

Foreign Ions	Added as	Amount Added (µg)	% Recovery	RSD (N = 3)
Cl^−^	NaCl	8.5 × 10^3^	98.5	1.69
Br^−^	NaBr	9.0 × 10^3^	100.0	2.48
PO_4_^2−^	Na_2_HPO_4_	5.0 × 10^3^	95.0	3.65
NO_3_^−^	NaNO_3_	2.5 × 10^3^	100.0	3.30
CO_3_^2−^	Na_2_CO_3_	1.8 × 10^2^	100.0	2.97
SO_4_^2−^	Na_2_SO_4_	5.0 × 10^2^	95.5	1.95
Na^+^	NaCl	6.5 × 10^3^	99.0	2.50
K^+^	KCl	5.0 × 10^3^	100.6	1.56
Ca^2+^	CaCl_2_	2.0 × 10^3^	97.5	3.45
Mg^2+^	MgCl_2_	2.0 × 10^3^	98.0	1.68
Zn^2+^	ZnCl_2_	2.5 × 10^2^	98.4	1.15
Cd^2+^	CdCl_2_	1.0 × 10^2^	98.5	4.3
Ni^2+^	NiNO_3_	3.0 × 10^2^	99.8	4.0
Cu^2+^	CuNO_3_	2.5 × 10^2^	98.0	3.68
Co^2+^	CoNO_3_	2.5 × 10^2^	96.8	4.26
Humic acid	C_6_H_9_NO_6_	50	94.5	3.87
Fulvic acid	C_12_H_12_O_8_	35	94.0	3.65

**Table 3:** Analytical method validation of the proposed methodology.

Samples	Certified Value (µg g^−1^)	Value Found by Proposed Method (µg g^−1^) ^a^ ± Standard Deviation	Value of *t*-Test ^b^
NISTSRM 1641d	1.56 ± 0.02	1.52 ± 0.04	1.36

^a^ Mean value, N = 3; ^b^ at 95% confidence level.

**Table 4:** Analyses of real samples after solid phase extraction using GO/CdS and ICP-OES determination.

**Samples**	**Amount Added (µg)**	**Hg(II) Found (µg L^−1^) ± Standard Deviation ^a^**	**Recovery Percent of Added Amount** **(RSD)** **^c^**	**Value of *t*-Test ^d^**
Tap water	0	ND ^b^	-	-
	5	5.05 ± 0.24	101 (0.18)	0.78
	10	9.92 ± 0.46	99.2 (0.22)	1.18
River water	0	5.5	-	-
	5	10.60 ± 0.35	102. (0.35)	1.28
	10	15.56 ± 0.82	100.6 (0.32)	1.45
Groundwater	0	1.30 ± 0.45	-	1.41
	5	6.28 ± 0.15	99.6 (0.28)	1.94
	10	11.32 ± 0.52	100.2 (0.36)	2.41

^a^ N = 3; ^b^ not detected; ^c^ Relative standard deviation; ^d^ at 95% confidence level, *t*_critical_ = 4.303.

**Table 5:** Comparative data of analytical features of the reported adsorbents.

Adsorbent	Sorption Capacity (mg g^−1^)	Method	Detection Limit (µg L^−1^)	Ref.
MoS_2_ nanocomposite	160.4	SPE-icpoes	0.09	[1]
Magnetite carbon/dithizone nanocomposite	-	SPE-voltammetry	2.7	[13]
Magnetic MoS_2_ nano-hybrid	428.0	SPE	3.2	[23]
Metal–organic framework	1600	Dispersive SPE-icpoes	-	[14]
Cellulose/ZrO_2_	180	SPE-icpoes	0.05	[36]
GO/CdS	186.0	SPE-icpoes	0.07	This work

## Data Availability

Not applicable.
